# Aortic stiffness and blood pressure variability in young people: a multimodality investigation of central and peripheral vasculature

**DOI:** 10.1097/HJH.0000000000001192

**Published:** 2016-12-05

**Authors:** Henry Boardman, Adam J. Lewandowski, Merzaka Lazdam, Yvonne Kenworthy, Polly Whitworth, Charlotte L. Zwager, Jane M. Francis, Christina Y.L. Aye, Wilby Williamson, Stefan Neubauer, Paul Leeson

**Affiliations:** a Oxford Cardiovascular Clinical Research Facility; b Oxford Centre for Clinical Magnetic Resonance Research, Division of Cardiovascular Medicine, Radcliffe Department of Medicine, University of Oxford, Oxford, UK

**Keywords:** aortic stiffness, blood pressure variability, pulse wave velocity, young adults

## Abstract

**Introduction:**

Increased blood pressure (BP) variability is a cardiovascular risk marker for young individuals and may relate to the ability of their aorta to buffer cardiac output. We used a multimodality approach to determine relations between central and peripheral arterial stiffness and BP variability.

**Methods:**

We studied 152 adults (mean age of 31 years) who had BP variability measures based on SD of awake ambulatory BPs, 24-h weighted SD and average real variability (ARV). Global and regional aortic distensibility was measured by cardiovascular magnetic resonance, arterial stiffness by cardio-ankle vascular index (CAVI) and pulse wave velocity (PWV) by SphygmoCor (carotid–femoral) and Vicorder (brachial–femoral).

**Results:**

In young people, free from overt cardiovascular disease, all indices of SBP and DBP variability correlated with aortic distensibility (global aortic distensibility versus awake SBP SD: *r* = −0.39, *P* < 0.001; SBP ARV: *r* = −0.34, *P* < 0.001; weighted 24-h SBP SD: *r* = −0.42, *P* < 0.001). CAVI, which closely associated with aortic distensibility, also related to DBP variability, as well as awake SBP SD (*r* = 0.19, *P* < 0.05) and weighted 24-h SBP SD (*r* = 0.24, *P* < 0.01), with a trend for SBP ARV (*r* = 0.17, *P* = 0.06). In contrast, associations with PWV were only between carotid–femoral PWV and weighted SD of SBP (*r* = 0.20, *P* = 0.03) as well as weighted and ARV of DBP.

**Conclusion:**

Greater BP variability in young people relates to increases in central aortic stiffness, strategies to measure and protect aortic function from a young age may be important to reduce cardiovascular risk.

## INTRODUCTION

Average levels of blood pressure (BP) for an individual are powerful indicators of cardiovascular risk [1]. More recently, measure-to-measure variability of that BP level has been identified as an additional, independent predictor of risk both for individuals with cardiovascular diseases [2–5] as well as young [6] and healthy populations [7,8]. Epidemiological evidence supports a lower disease burden in later life in those whose BP is better controlled during early adulthood [9]. BP variability may be of particular importance as increased variability appears to be a stronger marker of risk in younger than older individuals [3].

Greater reductions in cardiovascular endpoints with use of certain antihypertensive medications, despite similar BP reductions, may be because of their specific impact on BP variability [2]. There are likely to be multifactorial determinants of variability, which could provide targets for intervention, including baroreceptor sensitivity [10–13] and sympathetic activation [14–16], but aortic stiffness early in life [17] would be expected to be of specific relevance. The ability of the aorta to buffer pulsatile cardiac output (*CO*) and dissipate excess kinetic energy is a key component in BP regulation.

Peripheral measures of arterial stiffness such as pulse wave velocity (PWV) are associated with BP variability in older cohorts with hypertension and diabetes [18–20] but studies in young healthy individuals have been less consistent [6,21]. This may be because of heterogeneity in arterial stiffness between peripheral and central circulations earlier in life [22,23]. Comprehensive multimodality approaches to quantify both central and peripheral vascular measures in the same individual have allowed in-depth analysis of associations between clinical measures and arterial pathophysiology [24–27]. We therefore used this approach to test the hypothesis that BP variability in a large group of young individuals specifically relates to changes in aortic function.

## METHODS

### Study population

We studied 152 participants, aged between 20 and 49 years, who were clinically well and free from diabetes and overt cardiac, cerebrovascular and renal disease. All had been recruited to research studies at the Cardiovascular Clinical Research Facility and had undergone cardiovascular magnetic resonance (CMR) and noninvasive peripheral measures of arterial stiffness. All studies were approved by a local research ethics committee, and all participants provided written consent. Equivalent imaging, arterial measure and cardiovascular risk assessment protocols were used for all participants [28–30]. Briefly, to assess risk profile, participants attended a research clinic for BP, heart rate and anthropometry measures. Medical and lifestyle information was assessed by questionnaire [31,32] and blood samples collected following a minimum 6-h fast, centrifuged and separated for storage at −80 °C prior to analysis. Fasting lipid profiles and metabolic measures were measured at the John Radcliffe Hospital (Oxford, UK) biochemistry laboratory using validated clinical assays.

### Measurement of blood pressure variability

At the end of the study visit, participants were fitted with an appropriately sized BP cuff around their left arm, which was connected to a calibrated ambulatory BP monitor (TM-2430; A&D Instruments, Abingdon, UK) and worn for 24 h. Measurements were taken every 30 min during the day and every 60 min during the night. Daytime was defined as between 0700 and 2300 h and night-time as between 2300 and 0700 h. Participants self-reported the timing of their sleep to allow accurate discrimination of awake and sleep periods. Normal daily activities were encouraged, with participants asked to keep their left arm relaxed and still when measurements were taking place. The reading, editing and analysis of BPs were done using ABPM Data Analysis Software for Windows (version 2.40; A&D Instruments, Abingdon, UK). BPs were extracted to calculate SD of SBP and DBP during the awake period as well as weighted SD over 24 h, calculated with the following formula: 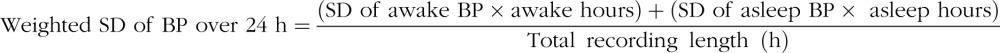


Average real variability (ARV) was calculated using the following formula: 
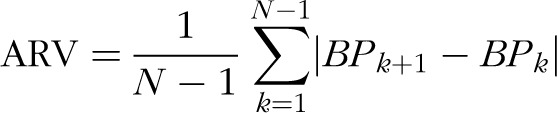


### Measurement of central aortic stiffness

#### Cardiovascular magnetic resonance

Aortic distensibility was measured on a 1.5-T scanner (Siemens, Munich, Germany) using a breath hold, ECG-gated, steady-state free precession sequence with a temporal resolution of 10 ms per cine frame. Sequences were applied to measure distensibility in the thoracic aorta at the level of the pulmonary artery, both in the ascending aorta and proximal descending aorta (PDA), respectively; in addition, in the distal descending aorta (DDA), 12 cm distal to the proximal descending aorta perpendicular to the lumen and in the abdominal aorta (AbA) at the level of the second lumbar vertebra. Compliance was measured using semiautomated edge detection algorithms to measure luminal area change (Matlab, Mathworks Inc., Natick, Massachusetts, USA). Distensibility was calculated by dividing compliance by central pulse pressure measured by Vicorder (Skidmore Medical, Taunton, UK) at time of magnetic resonance scan. A global measure of aortic distensibility was calculated from the mean value of distensibility measured in the ascending aorta, PDA, DDA and AbA.

### Measurement of peripherally obtained arterial stiffness

#### Cardio-ankle vascular index

CAVI was measured using VaSera VS-1500 (Fukuda Denshi Co. Ltd, Tokyo, Japan). CAVI is an indicator of the arterial stiffness from the origin of the aorta to the ankle. The measure is developed from the stiffness parameter β and is independent of BP at time of measurement [33,34].

#### Pulse wave velocity

Carotid–femoral PWV was measured using applanation tonometry to obtain pressure waveforms of the carotid and femoral pulse (SphygmoCor; AtCor Medical, West Ryde, Australia). Brachial–femoral PWV was measured using sphygmomanometer-derived indices (Vicorder, Skidmore Medical, Taunton, UK) with cuffs placed around brachial and femoral arteries to identify pulse arrival. With both techniques, PWV was derived from recording of the time delay between the two measurement sites, relative to the distance between them and identified from predefined landmarks.

#### Carotid and brachial distensibility

Carotid and brachial vessels were imaged using a Philips CX50 ultrasound machine (Philips, Andover, Massachusetts, USA) with 12-MHz linear array transducer. The participant lay supine in a temperature-controlled room and, following 10-min rest, ECG-gated longitudinal image loops of the right brachial artery were acquired 5–10 cm above the antecubital fossa The carotid arteries were imaged with patient lying flat and the head rotated to the opposite side from the carotid measure. The carotid bifurcation was identified, and then a longitudinal image loop acquired that included the bulb and common carotid artery so that measures could be performed 1 cm proximal to the bifurcation. Minimum and maximum arterial diameter of the carotid and brachial artery were measured offline from stored ultrasound image loops acquired over multiple cardiac cycles. Carotid and brachial diameters were measured using automated image analysis software (Vascular Analyser; MIA, Coralville, Iowa, USA) and distensibility of these arteries quantified as change in diameter relative to minimum diameter proportional to pulse pressure based on central and peripheral measures, respectively, recorded during the ultrasound image acquisition [35].

### Statistical analysis

Statistical analysis was carried out using SPSS version 22 (IBM, Armonk, New York, USA). Normality was assessed using Shapiro–Wilk test and visual assessment of histograms. Comparison between two groups for continuous variables for normally distributed data was performed using a two-sided, independent-samples student *t* test and Mann–Whitney test for nonparametric data. Comparisons between more than two groups were performed using analysis of variance test. Results are presented as mean ± SD. *P* values less than 0.05 were considered statistically significant. Correlations were assessed by Pearson tests for normally distributed data and Spearman tests for nonparametric data. To further explore relationships, nonlinear models were tested and the dataset was dichotomized according to high and low global aortic distensibility to see if any associations present in the total cohort were still present across the spectrum of aortic distensibility.

## RESULTS

### Study population

Demographics of the study population are presented in Table 1. All were aged less than 50 years and were free of overt cardiovascular disease. There was a slightly higher proportion of women (68%), and 16% were smokers. Mean levels of BP, lipid subfractions and HOMA indices were within normal ranges. Few were obese with average BMI within the normal range.

**TABLE 1:** Demographics and cardiovascular risk factors

Demographics	Value ± SD
*N*	152
Age (years)	31 (range: 20–49)
Male [*n* (%)]	49 (32)
Smokers [*n* (%)]	24 (16)
Height (m)	1.70 ± 0.09
Weight (kg)	70.0 ± 12.4
BMI (kg/m^2^)	24.1 ± 3.9
HOMA IR	0.72 ± 0.34
Total cholesterol (mmol/l)	4.40 ± 0.88
HDL (mmol/l)	1.47 ± 0.43
SBP (mmHg)	117.4 ± 12.7
DBP (mmHg)	72.8 ± 9.1
Mean arterial pressure (mmHg)	87.6 ± 9.6
Pulse pressure (mmHg)	44.6 ± 8.9
Heart rate (beats/min)	59.6 ± 8.5

Mean ± SD or range. HOMA IR, homeostatic model of insulin resistance; HDL, HDL cholesterol.

### Blood pressure variability and central versus peripheral measures

**FIGURE 1:**
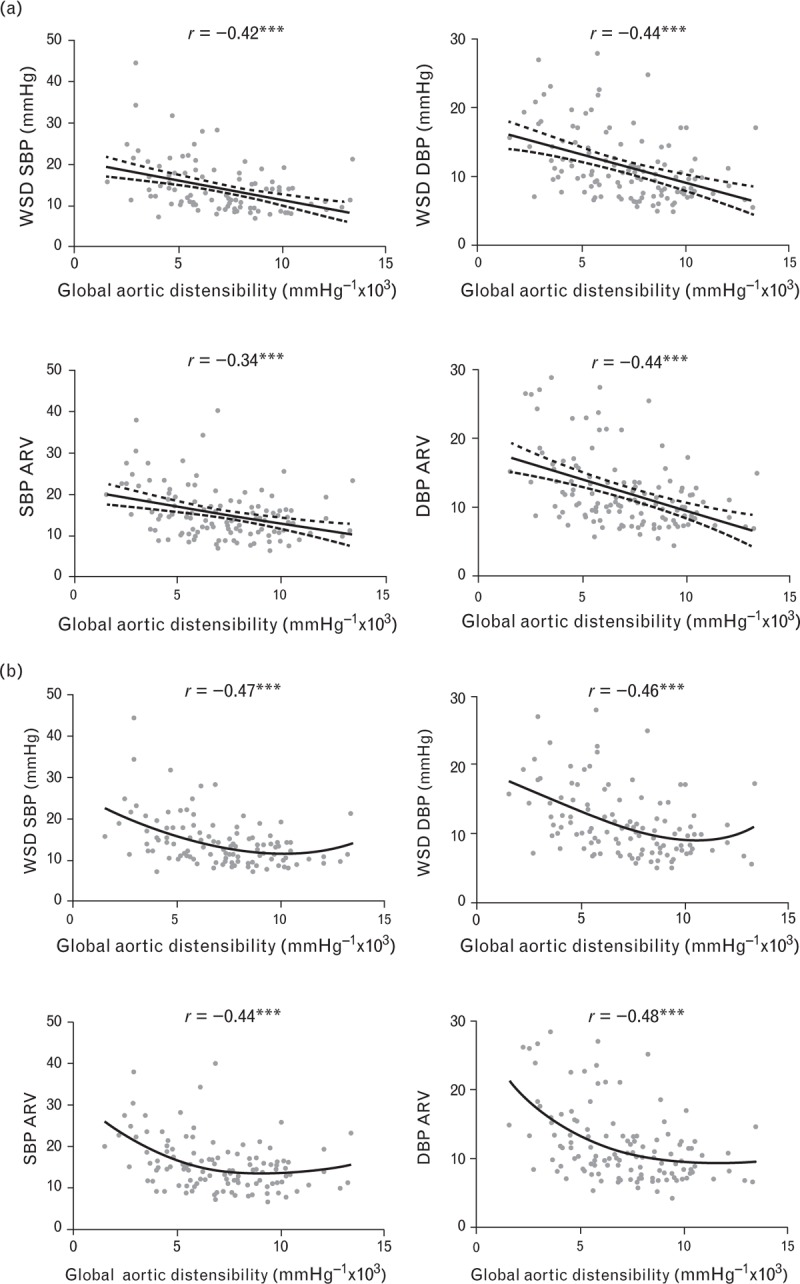
Relations between blood pressure variability and global aortic distensibility. ARV, average real variability, WSD, weighted 24-h SD. (^∗∗∗^*P* ≤ 0.001; ^∗∗^*P* ≤ 0.01; ^∗^*P* < 0.05). Panel (a): linear analysis. Panel (b): nonlinear analysis using cubic model (*Y* = *b*0 + (*b*1 × *X*) + (*b*2 × *X*^2^) + (*b*3 × *X*^3^).

Table 2 shows average ambulatory BP measures within the cohort. Average levels were within normal ranges, and BP variability values were consistent with previous reports in similar-aged populations. Table 3 shows that all indices of measure-to-measure BP variability had small but significant correlations with global measure of aortic distensibility for both SBP (SD awake SBP: *r* = −0.39, *P* < 0.001; weighted 24-h SBP SD: *r* = −0.42, *P* < 0.001; SBP ARV: *r* = −0.34, *P* < 0.001) and DBP (SD awake DBP: *r* = −0.39, *P* < 0.001; weighted 24-h DBP SD: *r* = −0.44, *P* < 0.001; DBP ARV: *r* = −0.41, *P* < 0.001). Small associations were also evident with aortic distensibility evaluated at each level of the aorta from ascending to abdominal aorta. The associations between the indices of SBP variability and global aortic distensibility are demonstrated in Fig. 1.

**TABLE 2:** Measures of blood pressure variability and arterial distensibility

Arterial parameters	Value ± SD
Average 24-h SBP (mmHg)	118.2 ± 9.3
Awake SBP (mmHg)	122.1 ± 9.9
SD SBP awake (mmHg)	15.0 ± 6.3
Weighted SD SBP (mmHg)	14.2 ± 5.8
ARV SBP (mmHg)	15.2 ± 6.1
Average 24-h DBP (mmHg)	70.3 ± 8.7
Awake DBP (mmHg)	73.8 ± 6.7
SD DBP awake (mmHg)	13.1 ± 6.0
Weighted SD DBP (mmHg)	11.5 ± 4.8
ARV DBP (mmHg)	11.8 ± 5.4
Aortic stiffness	
Global distensibility (mmHg^−1^×10^3^)	7.4 ± 2.7
Ascending aortic distensibility (mmHg^−1^×10^3^)	7.0 ± 3.0
Proximal descending aortic distensibility (mmHg^−1^×10^3^)	6.0 ± 1.8
Distal descending aortic distensibility (mmHg^−1^×10^3^)	8.8 ± 3.1
Abdominal aortic distensibility (mmHg^−1^×10^3^)	7.7 ± 3.6
Arterial stiffness	
CAVI	6.2 ± 0.8
PWV (carotid–femoral) (m/s)	5.7 ± 0.9
PWV (brachial–femoral) (m/s)	8.7 ± 1.5
Brachial distensibility (mmHg^−1^×10^3^)	1.18 ± 0.55
Carotid distensibility (mmHg^−1^×10^3^)	6.3 ± 2.2
Mean arterial pressure (mmHg)	87.6 ± 9.6
Pulse pressure (mmHg)	44.6 ± 8.9
Heart rate (beats/min)	59.6 ± 8.5

Mean ± SD or range. ARV, average real variability; CAVI, cardio-ankle vascular index; DDA, distal descending aorta; PWV, pulse wave velocity.

**TABLE 3:** Correlations between SBP and DBP variability and measures of aortic distensibility and stiffness

	SD of 24-h SBP – awake period	Weighted SD of 24-h SBP	SBP ARV	SD of 24-h DBP – awake period	Weighted SD of 24-h DBP	DBP ARV
Aortic distensibility						
Global	−0.39^***^	−0.42^***^	−0.34^***^	−0.39^***^	−0.44^***^	−0.41^***^
AA	−0.38^***^	−0.40^***^	−0.30^***^	−0.34^***^	−0.38^***^	−0.36^***^
PDA	−0.33^***^	−0.31^***^	−0.24^**^	−0.33^***^	−0.35^***^	−0.33^***^
DDA	−0.33^***^	−0.37^***^	−0.28^***^	−0.36^***^	−0.40^***^	−0.35^***^
AbA	−0.42^***^	−0.44^***^	−0.41^***^	−0.41^***^	−0.46^***^	−0.45^***^
Peripheral measures of stiffness						
CAVI	0.19^*^	0.24^**^	0.17	0.25^**^	0.31^***^	0.28^***^
cf PWV	0.11	0.20^*^	0.17	0.11	0.21^*^	0.21^*^
bf PWV	0.10	0.11	0.10	0.12	0.14	0.10
Peripheral distensibility						
Carotid	0.15	0.17	0.23^**^	0.19^*^	0.19^*^	0.23^**^
Brachial	−0.07	0.03	0.20	−0.16	−0.16	−0.07

AA, ascending aorta; AbA, abdominal aorta; bf PWV: brachial-femoral pulse wave velocity, Vicorder; CAVI, cardio-ankle vascular index; cf PWV, carotid-femoral pulse wave velocity, SphygmoCor; DDA, distal descending aorta; PDA, proximal descending aorta.

^*^P < 0.05.

^**^P ≤ 0.01.

^***^P ≤ 0.001.

Further testing of the associations between BP variability and global aortic distensibility using nonlinear models demonstrated that overall the strength of the association was similar to linear models [Fig. 1b shows the nonlinear model (cubic) with the best fit]. However, visually there did appear to be a greater increase in BP variability at lower levels of aortic distensibility. Therefore, we performed exploratory analysis to look at differences in gradient of association with the group divided based on whether individuals had lower global aortic distensibility (<5 mmHg^–1^ × 10^3^) compared with higher global aortic distensibility (≥5 mmHg^−1^ × 10^3^). The reduced sample size limited the power to test significance but different behaviour was evident with SBP ARV and DBP ARV (SBP ARV: −0.60, *P* = 0.01 compared with −0.18, *P* = 0.39; DBP ARV: −0.52, *P* < 0.05 compared with *r* = −0.25, *P* = 0.11), although consistent differences were not evident for other parameters (weighted 24-h SBP SD: *r* = −0.48, *P* = 0.12 compared with *r* = −0.32, *P* = 0.02; weighted 24-h DBP SD: *r* = −0.38, *P* = 0.32 compared with *r* = −0.32, *P* = 0.02).

Of the peripherally obtained measures, CAVI demonstrated small but significant correlations with several SBP variability indices including SD of awake SBP (*r* = 0.19, *P* < 0.05) and weighted 24-h SBP (*r* = 0.24, *P* < 0.01) as well as all indices of DBP variability (SD awake DBP: *r* = 0.25, *P* < 0.01, weighted 24-h DBP SD: *r* = 0.31, *P* < 0.001; DBP ARV: *r* = 0.28, *P* < 0.001) (Table 3). Carotid–femoral PWV assessed by SphygmoCor showed a small but significant correlation with weighted SD of both SBP (*r* = 0.20, *P* < 0.05) and DBP (*r* = 0.21, *P* < 0.05) as well as DBP ARV (*r* = 0.21, *P* < 0.05). In contrast, neither brachial–femoral PWV assessed by Vicorder nor brachial distensibility correlated with any of the BP variability measures. There was a small but significant correlation between carotid distensibility and SBP ARV (*r* = 0.23, *P* < 0.01) as well as indices of DBP variability (SD awake DBP: *r* = 0.19, *P* < 0.05; weighted 24-h DBP SD: *r* = 0.19, *P* < 0.05; DBP ARV: *r* = 0.23, *P* < 0.01) consistent with increased BP variability being associated with increased carotid distensibility.

### Relations between peripheral and central measures of arterial stiffness

**FIGURE 2:**
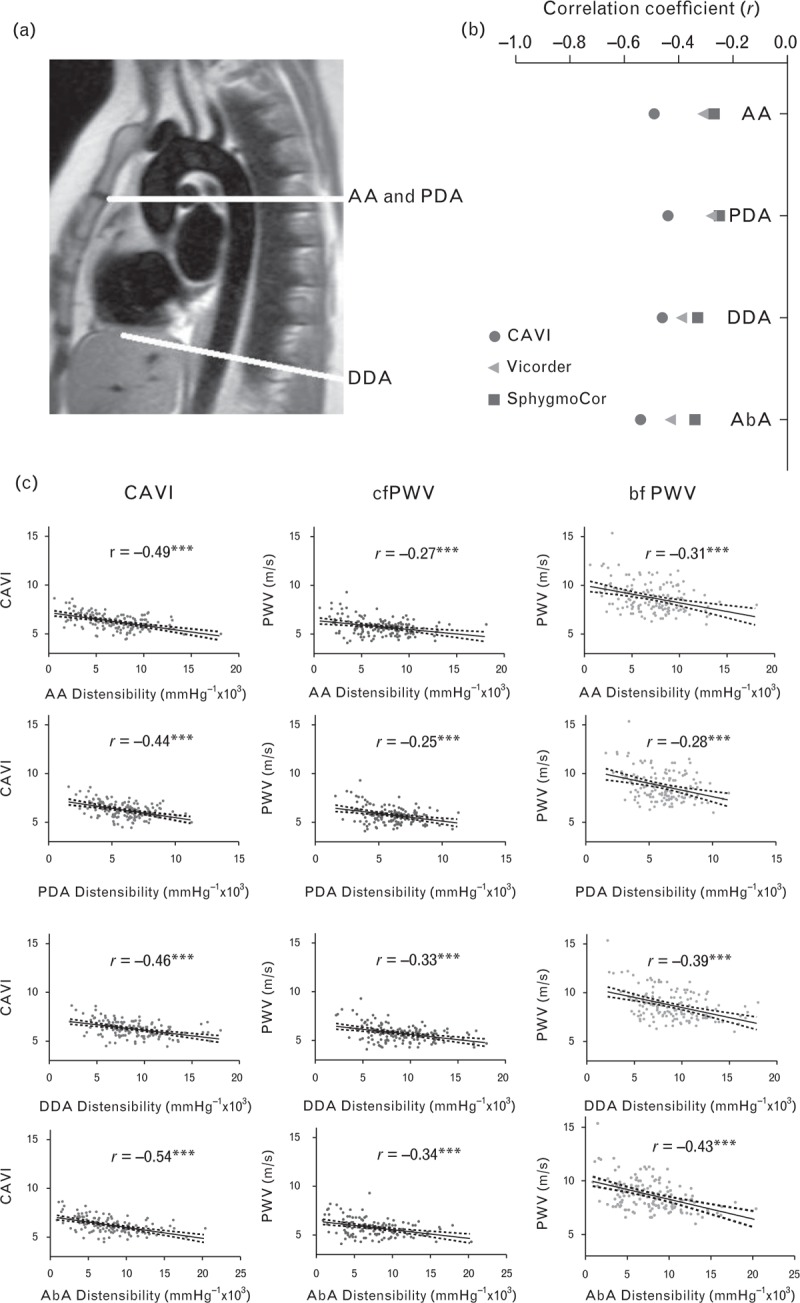
Correlations between central aortic distensibility and peripherally obtained measures of arterial stiffness. Cardio-ankle vascular index is most closely correlated with central aortic distensibility with a consistent association across the range of levels of aortic distensibility [part (b) (circle)] with weaker associations between central aortic distensibility and both carotid–femoral pulse wave velocity and brachial–femoral pulse wave velocity (triangle and square, respectively). (^∗∗∗^*P* ≤ 0.001; ^∗∗^*P* ≤ 0.01; ^∗^*P* < 0.05).

**FIGURE 3:**
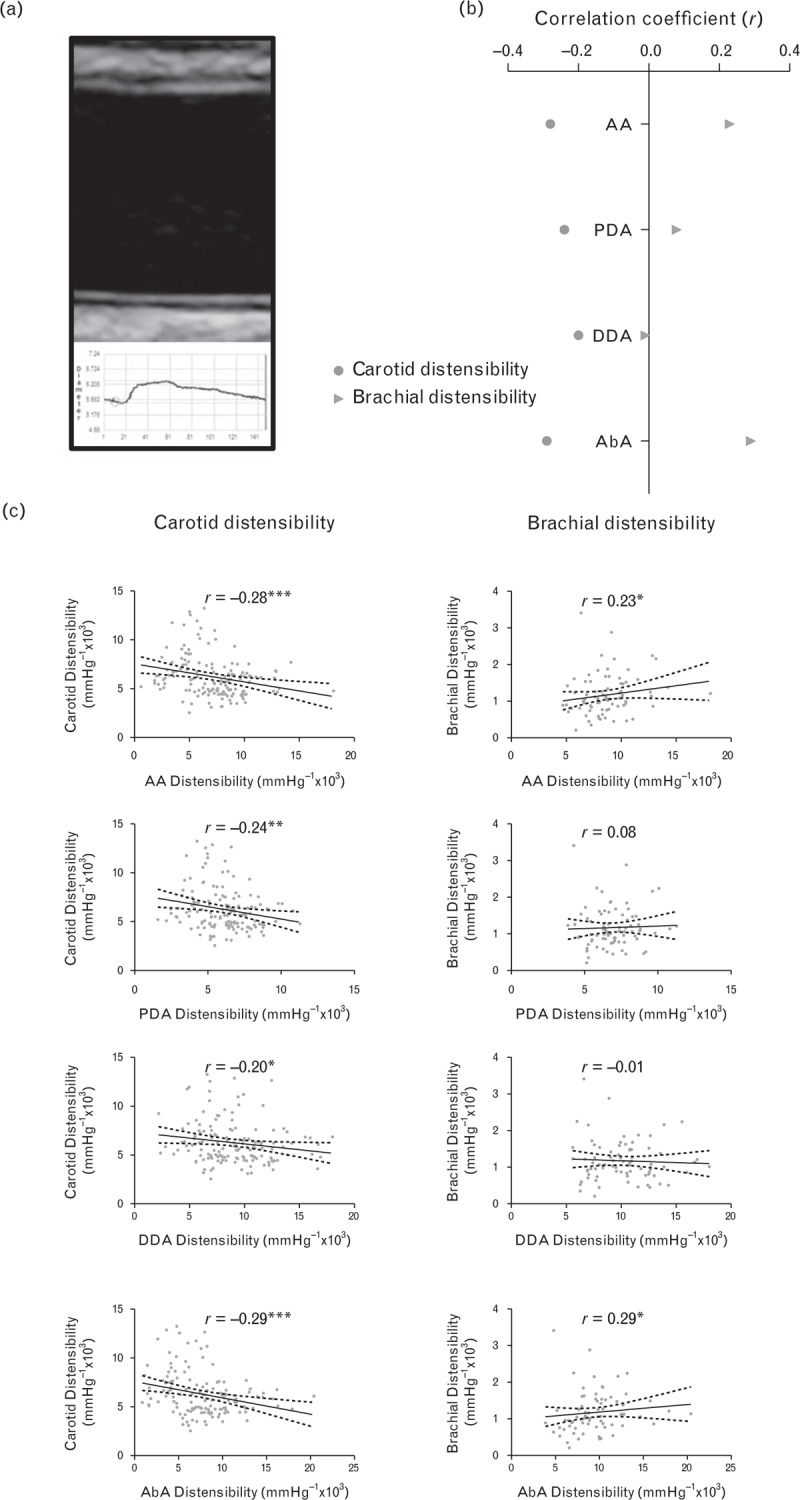
Correlations between central aortic distensibility and peripheral measures of arterial stiffness. Carotid distensibility was inversely correlated with aortic distensibility with consistent association across a range of levels of aortic distensibility [part (b) (circle)]. However, associations between brachial distensibility and aortic distensibility were weaker and inconsistent across different regions (triangle). (^∗∗∗^*P* ≤ 0.001; ^∗∗^*P* ≤ 0.01; ^∗^*P* < 0.05).

Figure 2 shows that the different measures of central and peripherally obtained arterial stiffness were significantly related to each other. However, those with the closest association with central aortic stiffness, measured by CMR, were those most closely related to BP variability. Global measures of aortic distensibility measured by CMR were significantly related to CAVI (*r* = −0.51, *P* < 0.001) with weaker correlations with PWV measured by SphygmoCor (*r* = −0.33, *P* < 0.001) and Vicorder (*r* = −0.40, *P* < 0.001). Similar patterns of association were seen with regional aortic distensibility at all four aortic levels, although associations were closer between PWV measured by SphygmoCor and Vicorder in the descending and abdominal aorta. Correlations between aortic and brachial distensibility were weak, as shown in Fig. 3, with only small but significant positive associations between brachial distensibility and aortic distensibility in the ascending and abdominal aorta, though not in the proximal or DDA. However, carotid distensibility demonstrated a small but significant inverse relationship with aortic distensibility at all four levels measured, with greater aortic distensibility being associated with reduced carotid distensibility.

## DISCUSSION

The current study shows that in young people, free from cardiovascular disease, increased BP variability associates with increased central aortic stiffness, while being less dependent on the distensibility of smaller conduit vessels, such as carotid arteries.

To our knowledge, this is the first study of its size to study BP variability in relation to a comprehensive multimodality assessment of arterial stiffness, including CMR, in a young adult population. Risk factors, such as hypertension, are underdiagnosed and undertreated in young adults, with up to one in 17 under 40-year-olds thought to be hypertensive [36]. Early adulthood is a time when stratification of cardiovascular risk through surrogate measures such as arterial stiffness and BP variability may be of particular value to identify those at greatest risk, who may benefit from targeted therapeutic intervention. Our findings of general associations between BP variability and arterial stiffness in young people extend findings from older populations. Schillaci *et al.*[18] showed BP variability correlated with carotid–femoral PWV in an older hypertensive population (mean age 49 ± 11 years), and Masugata *et al.*[37] demonstrated similar associations in a cohort 20 years older using CAVI to assess arterial stiffness (*r* = 0.33, *P* = 0.01).

The most substantial data on a specific link between BP variability and central aortic measures come from the Multi-Ethnic Study of Atherosclerosis of adults aged over 45 years. In this study, there were significant differences in SD SBP between the highest and lowest quartile of aortic distensibility [38]. Although there are several mechanisms that influence BP variability, the ability of the aorta to effectively buffer the pulsatile *CO* and dissipate excess kinetic energy is a key component in regulating fluctuations in BP. In individuals with reduced aortic compliance, BP is likely to be more affected by changes in *CO* and contractility, which might be altered by fluid status, posture and preload. We found that CMR measures that specifically quantify distensibility of the aorta were significantly related to all measures of BP variability. Peripheral noninvasive measures that incorporate central aortic segments in their evaluation of arterial stiffness were also associated with some aspects of BP variability. The most closely associated was CAVI, a global measure of arterial stiffness, which is considered to include assessment of the ascending aorta and arch in its output. CAVI was also most closely associated with CMR measures of central aortic stiffness. These findings are consistent with Horinaka *et al.*[39] who reported significant correlations between CAVI and stiffness parameter β in the ascending and descending aorta measured using ECG-gated multidetector row computed tomography (*r* = 0.49, *P* < 0.001 and *r* = 0.30, *P* = 0.03, respectively).

PWV measured from carotid to femoral arteries has a significant aortic component to its estimate of arterial stiffness, and the MARK study [40] has previously reported a correlation (*r* = 0.56, *P* < 0.01) between CAVI and carotid-to-femoral artery PWV in older populations (mean age 60.3 years). However, the measure is usually considered to exclude the ascending aorta and arch, and this was shown in our study in which associations between carotid–femoral PWV and regional aortic distensibility were strongest with the descending and abdominal aorta. Consistent with this reduced representation of proximal aortic stiffness, associations with BP variability were more limited. Brachial-to-femoral PWV (Vicorder) and specific measures of stiffness limited exclusively to the periphery, such as brachial artery distensibility, were unrelated.

Carotid artery distensibility in this population is of comparable magnitude to aortic distensibility; however, despite their similarities in structure [41], our data demonstrated an inverse relationship. Contrary to our data, it has been shown in older patients [42] and those with established coronary artery disease [43] that aortic and carotid distensibility are positively correlated. However, the relationship is not well explored in young individuals free from cardiovascular risk factors, and a similar inverse relationship has been reported in specific populations, such as pregnant women [44]. Compensatory reductions in stiffness of peripheral muscular arteries have been reported in hypertensive patients with increased central arterial stiffness [23]. Reduced ascending aortic and arch distensibility is likely to limit the Windkessel function of the aorta and lead to greater pulsatility and faster arrival time of *CO* to the carotid vessels. The degree of variability in vessel wall stress is likely to be accentuated in those with significant measure-to-measure BP variability, and it is possible that this initially leads to increases in distensibility within conduit vessels; a response that may be of particular importance to limit variability in cerebral perfusion.

An alternative explanation for the stronger correlations between CAVI and BP variability is that CAVI is independent of BP at time of measurement so that it indicates intrinsic arterial stiffness. CAVI utilizes stiffness parameter β, which is independent of BP at time of measurement, to derive an index of arterial stiffness [34]. PWV has a close and dependent relationship with BP [45], and therefore greater measure-to-measure variation in BP could lead to greater variation in the measure of PWV for an individual and to weaker correlation. However, carotid and brachial distensibility also take account of BP at time of measurement and differed in their relation with BP variability.

Associations with BP variability were evident across the range of stiffness measures. However, by exploring associations with nonlinear models, and at lower and high global aortic distensibility levels separately, our data suggest that the association may not be entirely linear. There appeared to be a more rapid increase in BP variability at lower levels of distensibility. Future studies in larger datasets will be of value to compare associations at different grades of aortic distensibility.

To improve the precision of our BP variability assessment, we used several techniques, including more recent measures such as weighted SD and ARV, in addition to SD of awake BP. These avoid the effects of night-time dipping on variability, which is a normal physiological phenomenon, but can give an inappropriate impression of adverse variability in measures that use whole 24-h SD of BP. A potential limitation is that our dataset was limited to measures of ‘short-term’ BP variability using 24-h ambulatory BP monitors. Some studies have shown that visit-to-visit (long-term) BP variability is of particular importance to cerebrovascular risk [2]. Nevertheless, a limited number of studies have compared measures head-to-head and suggest a good correlation between variability measured using visit-to-visit values and ambulatory BP monitors [3].

In this study, we focused on understanding the importance of central aortic stiffness versus peripheral muscular arterial tone to BP variability, and we did not consider the role of smaller resistance or microvessel function on BP variability nor neurological or other biological control. There was a slightly higher proportion of female participants in our study group, and it is known that sex may have specific effects on aortic function, potentially through hormonal factors [46], but we were underpowered to study subgroups separately. The assessment was also performed at a single time point and associative; therefore, we cannot determine the direction or causality of the relation between BP variability and arterial stiffness. It is possible that greater BP variability leads to accelerated vascular ageing, rather than reduced aortic function being a determinant of BP variability. Alternatively, the association may be circular. Future longitudinal and experimental studies will increase our understanding of the temporal and mechanistic relationship.

In summary, increased BP variability is closely associated with specific increases in central aortic stiffness in young people. Strategies to measure aortic stiffness and protect aortic function from a young age may be important to reduce cardiovascular risk.

## ACKNOWLEDGMENTS AND FUNDING

This work was supported by grants to P.L. from the British Heart Foundation (FS/06/024 and FS/11/65/28865). A.J.L. was supported by the Commonwealth Scholarship and Fellowship Program. H.B. was supported by an unrestricted grant from Fukuda Denshi (Tokyo, Japan) who manufacture VaSera. Additional grants were received from the National Institute for Health Research Oxford Biomedical Research Centre and Oxford British Heart Foundation Centre for Research Excellence.

H.B. discloses having received an unrestricted grant from Fukuda Denshi (Tokyo, Japan) who manufacture VaSera.

### Conflicts of interest

There are no conflicts of interest.

## Reviewer's Summary Evaluation

### Reviewer 1

It was the aim of this elegant investigation in 152 healthy participants, aged between 20 and 49 years, to test the hypothesis that blood pressure variability in a large group of individuals relates to the changes in aortic function. Blood pressure variability was correlated inversely with aortic distensibility. This was also true for the stiffness index CAVI. In contrast, the correlations between peripheral estimates of arterial stiffness (c-f PWV and b-f PWV) and aortic distensibility were very small, indeed. Especially with respect to the elegant direct measurements of central aortic distensibility in man this is an outstanding manuscript and I find it difficult to detect weaknesses in this study. It shows that greater blood pressure variability in young people relates to increases in central aortic stiffness. Strategies to measure and protect aortic function from a young age on may be important to reduce cardiovascular risk.
